# Validation of Brain Injury Guidelines in the Elderly Trauma Patient Presenting at a Level Two Trauma Center

**DOI:** 10.51894/001c.164359

**Published:** 2026-07-31

**Authors:** Sara N. Nesheiwat, Tarik Wasfie, Holland Korbitz, Jennifer Lynn Hille, Jennifer Hella, Kimberly Barber, Raquel Yapchai, Brian Shapiro

**Affiliations:** 1 Henry Ford Genesys Hospital

## 33

### Introduction

Traumatic brain injury (TBI) remains a major public health concern, accounting for approximately 2.5–2.8 million emergency department visits annually in the United States and contributing to significant morbidity, healthcare costs, unnecessary CTs, and inappropriate allocation of critical care resources. Among patients with mild TBI, up to 10–15% will have positive findings on head CT imaging, though only a small subset requires neurosurgical intervention. The Brain Injury Guidelines (BIG) were developed to help standardize the management of TBI patients with traumatic intracranial hemorrhage. These guidelines Risk-stratify patients based on CT findings, neurologic exam, and clinical risk factors. These guidelines were created to reduce unnecessary resource utilization while maintaining patient safety.

### Methods

A Retrospective analysis was used to validate the brain injury guideline (BIG) in the management of TBIs at a Level II trauma center. Patients with TBI’s were compared before and after implementation of the protocol. This included 542 patients seen in the Emergency Department with head injury between 2017 and 2021, divided into 2 groups: pre BIG protocol implementation (January 2017-June 2019) and post BIG protocol implementation (July 2019-December 2021.) Data collected included age, race, length of stay (hospital and ICU), comorbid conditions, anticoagulant therapy, surgical intervention, alcoholic intoxication (blood alcohol >80 mg/dL, GCS, findings of head CT, and any subsequent progression, mortality, and readmission within 1 month. Student’s t-test and Chi-square test were used for statistical analysis. Institutional Review Board approval was obtained before conducting the study.

### Results

There was a total of 314 patients in Group 1 (pre-BIG), 228 patients in Group 2 (post-BIG). The mean age of Group 1 was 59, and Group 2 was 67 years old. Groups 1 and 2 had no statistical difference in sex distribution. More patients in Group 2 had 4+ comorbid conditions than Group 1 (110,48% vs 78,25%, *p* = .0001.) Data on those that fit BIG 1 criteria was further analyzed and stratified. The post-implementation group was older, had more females and had a statistically significant number of patients with more than 4 comorbid conditions (29% vs 8%, *p* = .004.) The majority presented with <4 mm subdural or sub-arachnoid hematoma. No patient in either group had progression of their neurological examination, neurosurgical intervention, or readmission.

### Discussion

Despite the post-implementation group statistically being older and having more comorbidities. There were no re-admissions or worsening of brain bleeds when stratified into the BIG 1 group.

Observation in the ED often lasted longer than 6 hours due to medical management of disposition needs and increased the LOS if admitted to the hospital.

The Brain Injury Guidelines (BIG) provide a standardized, safety-focused framework for managing traumatic intracranial hemorrhage. By maintaining conservative thresholds for imaging, admission, and neurosurgical consultation, BIG minimizes the risk of missed progression and reduces variability in care. Its simple three-tier structure allows for consistent implementation across providers and institutions, supporting reliable and defensible clinical decision-making. Overall, serving the patient’s best interest while allocating resources appropriately.

